# Rethinking Gaming Disorder Prevention: A Socio-Ecological Model Based on Practitioner Insights

**DOI:** 10.3390/ijerph23010117

**Published:** 2026-01-17

**Authors:** Maya Geudens, Rozane De Cock, Bieke Zaman, Bruno Dupont

**Affiliations:** 1 Media, Culture & Policy Lab, Department of Communication Sciences, KU Leuven, 3000 Leuven, Belgium; rozane.decock@kuleuven.be (R.D.C.); bieke.zaman@kuleuven.be (B.Z.); bruno.dupont@kuleuven.be (B.D.); 2 Centre of Expertise Inclusive Society, UCLL Research & Expertise, UC Leuven-Limburg, 3001 Heverlee, Belgium

**Keywords:** gaming disorder, problematic gaming, prevention, socio-ecological model, practitioners, qualitative research, public health, digital media

## Abstract

**Highlights:**

**Public health relevance—How does this work relate to a public health issue?**

- Problematic gaming in adolescents is associated with mental health symptoms, sleep disruption, academic difficulties, and increased pressure on families and care services.
- Despite these concrete public health impacts, current prevention efforts remain fragmented and insufficiently embedded in existing youth and mental health systems. Prevention is hindered by specific gaps, such as unclear policy responsibilities, limited public awareness, and a lack of accessible guidance for parents, schools and frontline workers, underlining the need for coordinated action.

**Public health significance—Why is this work of significance to public health?**

- Drawing on in-depth interviews with prevention and early-intervention professionals, the study reveals structural barriers, including unstable funding, limited access to usable evidence, a shortage of practical tools, and difficulties engaging families with elevated risk. These barriers constrain effective early prevention across educational, clinical, and community settings.
- By organizing these barriers within a socio-ecological public health framework, the study clarifies how policy, institutional, community, and interpersonal factors interact, and identifies where improvements are most urgently needed to better support young people and their families.

**Public health implications—What are the key implications or messages for practitioners, policy makers and/or researchers in public health?**

- Policy makers should develop a coherent, long-term and sustainably funded multi-level, cross-sector prevention strategy that embeds gaming within broader digital well-being and youth mental health agendas, with clearly allocated responsibilities, stable budgets, and structural collaboration between education, youth care, mental health, and digital policy domains.
- Practitioners and researchers should co-create and evaluate evidence-informed, low-threshold tools and supports for institutions and families, using implementation science to strengthen adoption, adaptation and long-term sustainability in routine services and to better engage diverse youth and family populations.

**Abstract:**

Current approaches to gaming disorder prevention remain comparatively narrow, and prevention efforts are frequently underdeveloped and fragmented. Using the socio-ecological model (SEM), this qualitative study mapped frontline practitioners’ perceived obstacles and opportunities to develop a multi-level, practice-grounded framework for policy and implementation. Semi-structured interviews were conducted with 18 prevention professionals in Flanders (Dutch-speaking Belgium), recruited via purposive and snowball sampling. A hybrid inductive–deductive analysis—iterative coding guided by Layder’s adaptive theory—organized findings across SEM levels. At the public policy level, participants highlighted insufficient sustainable funding but saw potential in coordinated frameworks moving prevention beyond substance-focused agendas. At the community level, a clear knowledge gap emerged, with opportunities in integrating gaming within broader digital well-being efforts. Institutionally, the absence of practical tools and clear referral pathways was noted, in addition to high participation barriers, whereas accessible programs with targeted outreach were viewed as promising. Interpersonally, parental disengagement was common, but early involvement and pedagogical guidance were seen as key levers. At the intrapersonal level, limited self-insight and emotion regulation impeded change, while resilience, self-confidence, and offline activities were protective. This first empirical application of the SEM to gaming disorder prevention highlights the need for a multi-level, context-sensitive framework that bridges public health and digital media perspectives.

## 1. Introduction

Although much has been written about gaming disorder, prevention often remains an afterthought in both research and practice [1]. This study addresses a crucial question: how can the insights of prevention professionals, positioned at the intersection of multiple socio-ecological layers, guide us in effectively reducing problematic gaming behaviors? By situating their perspectives within the socio-ecological model (SEM), we propose a reframing of prevention as an ecosystem challenge rather than an individual problem.

Digital gaming has become one of the world’s most popular leisure activities, with an estimated 2.84 billion players in 2024 [2]. For most, gaming is enjoyable and adaptive, with cognitive, emotional, motivational, and social benefits such as improved attentional control and problem-solving, resilience after failure, mood enhancement, and prosocial behavior [3]. For a notable minority, however, excessive gaming can lead to serious challenges, including sleep disruption, academic underperformance, and mental health problems, including anxiety, depression, and even suicidal ideation [4,5,6,7]. A meta-analysis estimates that approximately 3% of individuals worldwide experience problematic gaming behaviors [8].

The impact extends far beyond individual players. The societal costs are significant: reduced productivity, increased healthcare expenditures, social isolation, a rise in divorces, and deteriorating family relationships [9,10,11]. This perception is reinforced by research emphasizing the need for more comprehensive and effective prevention strategies for problematic gaming [8,12,13]. This is not only felt within academia but also expressed by parents [14], professionals [15], and governments [16]. Preventive investment is also economically compelling: each dollar spent on preventing adolescent mental disorders yields substantial returns [17].

The urgency is undeniable: effective prevention strategies are needed to keep gaming healthy. Despite this urgency, prevention remains underdeveloped relative to treatment research [8]. Scientifically grounded interventions are scarce [18], and existing ones often focus on individual behaviors or school-based programs [19]. Rarely do they incorporate socio-ecological determinants—family dynamics, community norms, organizational practices, and policy instruments—that jointly shape gaming [20]. Addressing this gap requires a paradigm shift from siloed, individual-level solutions to coordinated, multi-level approaches.

We therefore adopt the socio-ecological model (SEM) as an integrative lens. In public health, the SEM conceptualizes health-related behaviors as shaped by interacting influences across multiple levels—typically including intrapersonal, interpersonal, institutional/organizational, community, and public policy determinants—rather than by individual factors alone [21,22]. It is widely used to structure multi-level prevention, including frameworks for violence prevention and adolescent substance-use prevention, as well as broader health promotion [23,24,25]. Yet it remains under-applied in gaming disorder prevention.

When applied to problematic gaming, the SEM maps how upstream structural conditions and downstream relational and individual processes jointly shape gaming behavior and prevention pathways. At the public policy level, broader socio-political frameworks—encompassing local, national, and international laws and regulations—shape the gaming environment; instruments such as age restrictions and regulations on in-game purchases have shown promise in reducing gaming-related harm [13]. At the community level, social and cultural norms, including prevailing perceptions of gaming and peer practices, play an important role in shaping gaming behavior [26]. At the institutional level, schools, community health centers, and other organizations affect individual trajectories: environments that promote well-being and provide structured activities can serve as protective factors, whereas failing to engage young people meaningfully may contribute to problematic digital behaviors [27]. At the interpersonal level, social relationships—including family dynamics, peer influences, and parental attitudes—are salient [28]. Finally, at the intrapersonal level, traits such as knowledge, attitudes, behaviors, impulsivity, and mental health conditions significantly shape gaming behaviors [29,30].

Guided by the SEM, a comprehensive approach that integrates multiple levels of influence and engages diverse stakeholders may provide a more effective and sustainable framework for preventing problematic gaming [31,32].

Prevention professionals are uniquely positioned to bridge the multiple layers of the socio-ecological system. They train intermediaries, develop tools, deliver early interventions, and contribute to policy initiatives, thereby moving between families, schools, community services, and governance structures [33]. While their involvement has been recognized as essential for understanding and addressing gaming problems [34], empirical research rarely places their perspectives at the center. Yet, their unique position affords them a system-wide view: they encounter the practical bottlenecks, cultural dynamics, and contextual conditions that determine whether preventive strategies remain theoretical or become actionable. Studies have examined provider perceptions in assessment and treatment contexts [35], but prevention-focused insights remain largely absent. This gap is surprising given their central role in the practical implementation of prevention programs.

To address these gaps, this study centers on the following research question: What opportunities and challenges do prevention professionals in Flanders (the Dutch-speaking northern part of Belgium) identify at the public policy, community, institutional, interpersonal, and intrapersonal levels regarding the prevention of problematic gaming behaviors?

## 2. Materials and Methods

### 2.1. Participants

A structured sampling strategy was employed to ensure a diverse group of participants, including prevention professionals, early intervention workers, and staff from expertise centers. The recruitment process aimed to achieve a balance in regional representation across Flanders as well as variations in roles and departments, such as prevention professionals from Mental Health Care Centers (MHCCs), municipal organizations, and federal agencies. Given the demanding schedules of these professionals, multiple recruitment pathways were utilized to ensure participation. First, recruitment began by reaching out to a working group of prevention professionals specifically focused on gaming. Second, a snowball sampling method was applied, where participants were encouraged to recommend others who could add value to the research. Third, gaps in regional and departmental representation were identified, prompting direct outreach to professionals from underrepresented provinces or sectors to ensure a well-rounded sample.

The final sample included 18 participants who often combined multiple roles:

- Prevention professionals (*n* = 15): education for parents and intermediaries, policy development in schools and health or welfare institutions.
- Early intervention workers (*n* = 11): short-term guidance (1–7 sessions) for youth with risky gaming behaviors and their parents.
- Addiction expertise center staff (*n* = 2): development and evaluation of science-based prevention methods for gaming, gambling, alcohol, and drugs.
- Media literacy coordinator (*n* = 1): management of a website promoting media literacy for parents.

A detailed overview of participant roles is provided in Supplementary Material S2.

### 2.2. Data Collection

Interview data were collected between June and November 2022. Each interview lasted between 60 and 90 min and was conducted in Dutch via video conferencing. The interviews were guided by a semi-structured interview guide, exploring participants’ experiences with problematic gaming and their views on effective prevention strategies. All interviews were audio-recorded with the participants’ consent and transcribed verbatim.

### 2.3. Procedure

This study employed a qualitative methodology to gain in-depth insights into the experiences and perspectives of prevention professionals. Semi-structured interviews were chosen for their flexibility, allowing both predefined themes and unexpected insights to be explored [36]. This study forms part of the SBO FWO Gam(e)(a)ble research project on gaming–gambling convergence (S006821N); therefore, the interview guide included dedicated questions on gaming–gambling convergence in addition to questions on problematic gaming prevention (see Supplementary Material S1). The present analysis focuses on prevention needs and implementation barriers.

### 2.4. Data Analysis

This study employed a hybrid inductive–deductive approach. Guided by Layder’s Adaptive Theory [37], we iteratively moved between inductive coding of participants’ accounts and theory-informed refinement, repeatedly checking interpretations against the original transcripts. Themes were anchored in the SEM level that best captured their primary emphasis, although determinants often exhibited cross-level linkages. Interview data were used to specify how each level manifested in gaming disorder prevention.

Data analysis and coding were conducted in Dutch on the verbatim transcripts; quotations were translated into English for reporting. Codes and interpretations were continually reviewed and consolidated using Saldaña’s [38] initial, axial, and pattern coding. Peer debriefing with colleagues helped refine interpretations and identify potential biases. Data were managed in NVivo 14.

### 2.5. Ethics

Ethical approval was obtained (G-2021-3439-P02(AMD)) by the Social and Societal Ethics Committee at KU Leuven on 25 October 2021. The study procedures were carried out in accordance with the Declaration of Helsinki. All participants were informed about the research objectives, methods, and their right to withdraw at any time without consequences, and all provided informed consent. To ensure confidentiality, identifying details were removed and replaced with generic descriptions (e.g., ‘prevention worker’). All authors of this study conduct research on gaming and gambling, which provides a deep understanding of the field but also necessitates critical self-reflection to mitigate potential biases. One of the researchers was actively involved in a prevention professional working group, overlapping with some participants. While this dual role facilitated trust-building and provided valuable insights into practical prevention work, it also introduced challenges related to power dynamics. To minimize any hierarchical influences, the interview guide was carefully designed to encourage open dialogue without implicit evaluation.

## 3. Results

**Figure 1:**
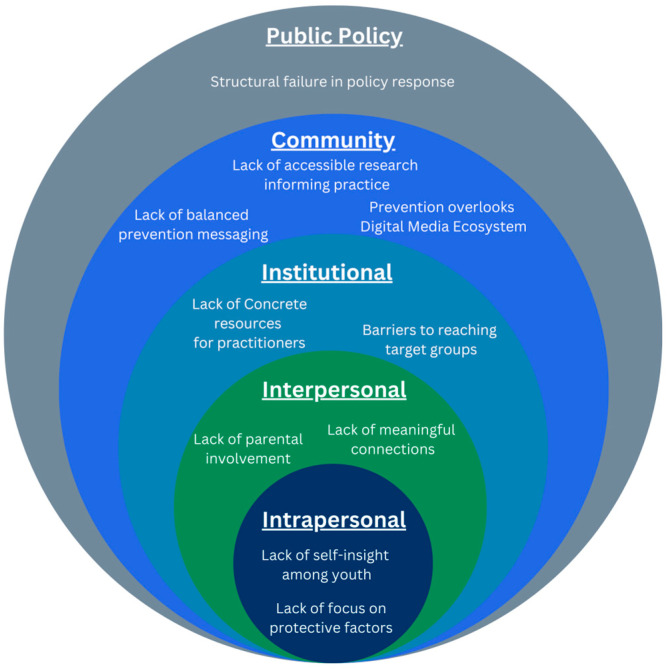
Socio-ecological framework for gaming disorder prevention. Key challenges are mapped onto the socio-ecological model, illustrating multi-level influences identified by prevention professionals.

Results are presented across SEM levels, progressing from public policy to community, institutional, interpersonal, and intrapersonal factors. For each theme, we first outline the main challenge, then explore potential causes and consequences, and describe proposed solutions. This macro-to-micro ordering foregrounds upstream determinants and illustrates how gaps cascade across levels. Figure 1 summarizes these findings.

### 3.1. Public Policy Factors

#### Structural Failure in Public Policy Responses

Prevention workers consistently indicate that current public policy frameworks at the regional and national levels fail to address problematic gaming in a structured and sustainable manner. Instead of dedicated policies and resources, gaming prevention is often implicitly added to addiction services focused on drugs and alcohol, leading to fragmentation, limited funding, and weak coordination. Participants pointed to different possible causes for this gap. Policymakers often lack awareness of the complexity and impact of problematic gaming. One participant explained: “Working towards policy, that’s often what’s missing. It’s about creating awareness, making people conscious—if it even gets through, right?” He added, “It’s one thing to raise awareness within policymakers, but convincing them to take action afterward? That’s a whole different step.” (P16). Additionally, participants said gaming disorder is often overlooked because it does not create immediate public disruptions, which typically drive swift policy responses.

The consequences are significant. Prevention workers noted that without dedicated resources, addiction services must absorb gaming prevention without extra support. “We often lack both the budget and the time to address this issue.” (P03) This also leads to a severe shortage of specialized services, resulting in long waiting lists and forcing young people to travel considerable distances for assistance. “People need to find their way, but for that, you need something to refer to, and that’s often missing.” (P02) This misalignment made many participants feel frustrated and left prevention programs underdeveloped and unable to adequately support the growing number of individuals seeking help. Participants also stressed the importance of proactive policymaking, with clear mandates, specialized services, and better coordination to ensure effective implementation.

### 3.2. Community Factors

At the community level, participants described challenges that partly overlap with broader digital well-being and addiction-prevention contexts (e.g., stigma and moralizing narratives), alongside issues more specific to gaming-related norms and literacy.

#### 3.2.1. Lack of Accessible Research Informing Practice

Participants highlighted a critical need for improved knowledge dissemination and awareness at all societal levels to address problematic gaming effectively. Although current research provides valuable evidence-based insights, it remains largely inaccessible in practice due to difficulties in locating and interpreting findings: “There is a lot of research, but you really have to look for it consciously. The average intermediary won’t do that. Accessible, readable research could make such a difference.” (P14)

Parents, teachers, and therapeutic professionals often feel ill-equipped to address gaming-related problems. Parents struggle with generational differences and lack of familiarity with gaming culture. One prevention worker explained: “Parents often think: in my day, we just played outside. Times have changed, but that mindset hasn’t.” (P07). Teachers, too, face difficulties: “Teachers notice the negative consequences in the classroom, like students falling asleep because they gamed all night. But they often react incorrectly, in a very accusatory way, because they don’t know what’s behind it.” (P02) Similarly, therapeutic professionals often view gaming as a specialized field outside their expertise.

The consequence is that parents and teachers frequently respond inappropriately, which can exacerbate gaming-related problems rather than mitigate them. Therapists, meanwhile, may avoid addressing gaming issues altogether: “They say, ‘I don’t know how to handle this,’ and as a result, it’s often ignored completely.” (P13)

Prevention workers proposed the development of evidence-based resources tailored to these groups.

#### 3.2.2. Lack of Balanced Prevention Messaging

Participants saw the absence of balanced prevention messaging as a major challenge. They noted that moralizing or fear-based approaches foster stigma and resistance, and argued instead for highlighting both the benefits and risks of gaming to promote constructive dialogue. “Parents tend to over-problematize gaming, which leads to faulty communication patterns that become unsolvable over time.” (P06) Similarly, participants reported that some professionals react from a place of panic: “Professionals are looking for a framework that clearly states that gaming is not okay. But the real work lies in reframing and addressing this topic with nuance.” (P12) Prevention workers warned that uncritical use of terms like ‘gaming addiction’ can pathologize normal play, increase stigma, and discourages help-seeking: “We often hear parents say, ‘He’s addicted to gaming,’ but when you ask further, he still goes to youth group and does well in school. Youngsters don’t like to be labeled as addicts.” (P01) At the same time, prevention workers acknowledged the value of an official diagnosis, as it helps prevent overdiagnosis. “If we have a clear diagnosis with conditions or specific criteria that must be met, I think that can be very helpful for prevention workers or when I give training. It allows us to say: ‘Look, these are the criteria—let’s not be too quick to call it gaming addiction.’” (P05)

Participants criticized the use of fear-based prevention messaging and called for a shift toward balanced communication that normalizes gaming as a common activity, while still educating about potential risks and encouraging healthy habits. This strategy, they argued, should be grounded in open dialogue that acknowledges gaming’s cognitive, social, and emotional benefits, and thus be more relatable and reduce stigma.

#### 3.2.3. Prevention Overlooks the Digital Media Ecosystem

Participants emphasized the need to expand prevention beyond gaming, advocating for a broader approach including social media, screen use, and digital well-being. The current, narrow focus fails to accurately reflect young people’s interconnected digital lives, where gaming, streaming, and social media coexist. “Parents often come to us with concerns about gaming, but their kids are also on social media or watching Netflix. They don’t always see how it’s all connected.” (P11) Another explained: “It’s always broader than just gaming. We need to open up this conversation.” (P12).

Several underlying factors contribute to this limited scope. First, prevention workers are often only funded for gaming-specific programs, leaving no resources to address broader digital behaviors. Second, gaming prevention is embedded within the substance addiction network. Prevention workers often address gaming alongside drugs and alcohol, with intervention programs frequently delivered in addiction centers. Individuals seeking help for gaming issues often share facilities and waiting areas with those struggling with substance abuse, reinforcing outdated frameworks.

This narrow focus has important consequences. Prevention efforts fail to engage a broader audience and often feel less relevant. “If you only talk about gaming, you lose a large part of the class.” (P13) It also limits attention to other digital behaviors, leaving issues like the convergence of gaming and gambling to be overlooked. Participants noted a widespread lack of awareness of this convergence among intermediaries, parents, youth, and even themselves. “Perhaps we need to be more conscious of asking about gambling too… This makes me realize… that’s something I don’t do either, because it’s not in the materials I use.” (P07)

In addition, stigmatization linked to outdated substance addiction frameworks further hinders help-seeking and reinforces misconceptions. Associating gaming with substance abuse can make individuals downplay their problems or avoid support altogether: “Because it’s placed under the category of alcohol and drugs, some people feel like, well, it’s not that bad… people don’t associate something like ‘the drugline’ [De Druglijn, a telephone helpline for people with drugs or alcohol issues] with gaming issues.” (P09)

Participants advocate a more integrated approach to prevention, such as the digital balance model developed by the Trimbos Institute, which promotes a healthy digital media balance, focusing on online-offline integration rather than just reducing screen time. “I am very drawn to the digital balance model because it doesn’t take a normative stance and offers a broader, more constructive view. That’s why I’ve shifted my focus from exclusively problematic gaming to also asking: ‘What constitutes a healthy digital diet?’” (P06)

### 3.3. Institutional Factors

#### 3.3.1. Lack of Concrete Resources for Practitioners

Participants repeatedly highlighted the shortage of practical tools and methodologies to support both professionals and parents. Many feel unsure about how to address gaming-related issues: “I sometimes really miss concrete methodologies on how to work with a young person around gaming.” (P07) Existing tools, according to participants, are too vague and fail to meet the needs of their target groups. Practitioners “long for something hands-on” (P05). This demand frequently surfaces during training sessions. As one noted: “That’s often the question we get during training: give us some guidelines, some concrete material.” (P13)

The lack of practical tools leaves professionals feeling uncertain. “We often don’t feel very confident; we really want some methodologies.” (P13) Participants expressed that “we still miss more concrete guidelines or tips because parents often don’t know what to do. This includes support in how to parent, how to give boundaries.” (P10) As one professional concluded, “If I could create one thing, it would be more concrete methodologies.” (P13)

#### 3.3.2. Barriers to Reaching Target Groups

Participants pointed to barriers in the accessibility, awareness, and practical implementation of existing prevention tools. They perceive a significant gap between the high perceived demand for prevention programs and the persistently low participation rates among target groups. “The demand is so high, and it’s disappointing; you develop something yet programs don’t fill up.” (P03)

Stigma, shame, financial constraints, and time pressures all limit engagement. Reaching specific groups—such as non-native speakers, culturally diverse families, and those with psychological or cognitive challenges—remains especially difficult. “The ones who need it the most… they are the hardest to reach.” (P07). An overload of online resources of varying quality further complicates navigation: “There are so many websites… How can anyone be expected to see the forest for the trees?” (P03). The lack of an integrated referral system further complicates directing people to the right support. “If we, as prevention workers, don’t know where to refer people, how can a parent or young person be expected to know?” (P08)

According to participants, these barriers leave vulnerable groups underserved. Participants stressed the urgency of developing low-threshold programs that are affordable, culturally sensitive, and easy to access. They also called for targeted outreach through local networks to better engage hard-to-reach populations. In addition, they advocated for a central, reliable platform that consolidates high-quality information and offers clear referral pathways for both online and offline support. As one prevention worker put it, “There is a need for an inventory of where people can go with questions and concerns.” (P05) Still, some warned against adding to the clutter: “You can make 10 more websites, if people don’t know it exists, it’s useless.” (P08)

### 3.4. Interpersonal Factors

#### 3.4.1. Lack of Parental Involvement Hinders Prevention

Participants overwhelmingly viewed parents as key figures in prevention and early intervention, some even as the most important stakeholders. “[Prevention] is deeply rooted in a parenting approach. Parents must be closely involved in this process.” (P13) However, they noted that many lack insight into their children’s online behavior and associated risks, such as exposure to gambling mechanics or unauthorized purchases: “parents often don’t know what their kids are doing online.” (P18) Another added: “The most significant problems arise in situations where parents are disengaged.” (P06) Another common theme was that parents underestimate their influence. “They often assume that because their child is a teenager and becoming independent, their role has diminished. But they still have a significant impact.” (P02)

Participants highlighted that many parents adopt a reactive approach, taking action only when problems escalate. One prevention worker described their frustration, saying, “It has to get out of hand before they seek help.” (P03) Early involvement was widely seen as essential. “It would be beneficial if you could get parents early… to introduce that critical voice, because children won’t do that themselves.” (P01) Another added: “We regularly aim to involve parents, as their role is pivotal.” (P05)

Participants advocated for providing parents with proactive strategies to address gaming-related risks before they escalate. Participants frequently pointed towards the need for pedagogical guidelines specifically tailored to help parents and caregivers address problematic gaming in children. They stressed that these guidelines should focus on establishing clear, consistent rules regarding game use, fostering open communication about gaming, and providing practical tools to supervise children’s gaming behavior effectively.

#### 3.4.2. Increasing Meaningful Connections

Fostering a sense of belonging, both within families and in wider social environments, was consistently identified as key to preventing problematic gaming. Participants stressed that genuine connections help young people build important skills and relationships, reducing their reliance on gaming. “For young people, it’s particularly important to feel connected to their surroundings.” (P15) Another added, “It is important to stimulate social life outside of gaming. If you become too isolated, it impacts your social and emotional development.” (P04)

Close familial bonds, such as shared activities or open communication, were seen as strong protective factors. Providing opportunities for meaningful offline engagement was considered essential to strengthening these connections and equipping youth with a well-rounded support system beyond the digital realm.

### 3.5. Intrapersonal Factors

#### 3.5.1. Lack of Self-Insight Among Youth

Participants noted that, like their parents, young individuals often fail to recognize the severity of problematic gaming behavior until it becomes acute. “Gamers only realize it as problematic when it is too late.” (P02) Participants suggested that this is partly because many underestimate their own habits, assuming others are worse. As one noted, “Young people think that others’ behavior is worse, which makes them think, ‘I’m not that bad…’ That correction of the social norm needs to happen.” (P01) This distorted perception often hinders critical self-reflection and help-seeking. “Youth neither ask questions nor look for information because they don’t think it’s a problem.” (P04) To address this, participants highlighted the need for programs that promote self-insight. These should raise awareness, help youth distinguish healthy from problematic gaming, and strengthen game and media literacy.

#### 3.5.2. Lack of Focus on Protective Factors

Prevention professionals emphasized the importance of addressing broader psychological and social influences in prevention efforts. Many noted that young people often struggle with emotion regulation, leading them to use gaming as a coping mechanism. “They need more self-esteem and confidence, along with better emotion regulation—an area where many gamers face significant challenges.” (P15) Others observed that many lack sources of fulfillment outside gaming. “Gamers often have nothing else they’re good at beyond gaming.” (P02)

Participants stressed the need to develop core personal and social skills—such as resilience, self-confidence, emotional regulation, and social competence—while also promoting meaningful offline activities. Encouraging alternative hobbies and interests was seen as essential to reducing problematic gaming and fostering a more balanced lifestyle.

## 4. Discussion

The question of how to effectively prevent gaming disorder remains largely unanswered, despite growing academic attention [8,39]. This paper addresses a key gap by centering the perspectives of prevention professionals and situating them within the SEM. Our findings emphasize the need for a multilevel, socio-ecological approach to gaming disorder prevention, addressing gaps at the policy, community, institutional, and interpersonal levels. By analyzing these challenges across the SEM, we propose an integrated framework for a more holistic and effective approach. The following sections present key takeaways and the solutions proposed by participants, highlighting actionable strategies across the SEM’s interconnected domains.

### 4.1. Implications of Structural Failure in Public Policy Responses

While gaming disorder is increasingly recognized as a public health issue [40], policy development remains slow [39]. Our findings show that this stagnation has tangible consequences: prevention professionals face resource shortages, time constraints, and vague policy directives, limiting the implementation of effective strategies. As a result, professional frustration persists, and individuals seeking help receive insufficient support. The study also highlights a critical gap in policymakers’ understanding of the complexity and societal impact of gaming disorder. This lack of awareness leads to underfunding and minimal prevention efforts. Participants noted that gaming-related issues are often deprioritized in favor of more visible concerns, such as substance abuse or crime. According to participants, addressing gaming disorder requires more than awareness-raising; it demands proper funding and a coherent prevention framework. In many Western countries, including Belgium, prevention is largely driven by non-profit organizations and private actors [5]. This decentralized approach results in fragmented initiatives that fail to comprehensively address the issue. Participants stressed the urgent need for a unified, evidence-based policy framework that incorporates best practices across sectors. Without structural reforms, gaming disorder will continue to be neglected within public health.

It may therefore be valuable to examine more systematized approaches abroad when considering how to strengthen prevention in Belgium. For example, South Korea is frequently cited as having government-supported prevention and counseling infrastructures [15], although evidence on the effectiveness of some restrictive measures is mixed—underscoring the importance of context-sensitive adaptation and rigorous evaluation when learning across settings [40].

### 4.2. Evidence-Based Knowledge Gap

Effective prevention requires that professionals—including prevention workers, teachers, general practitioners, and parents—are well-informed about gaming disorder [18]. Yet insufficient knowledge among professionals remains a major barrier [41]. Individuals with gaming problems often perceive healthcare providers as inadequately trained, which discourages help-seeking [42].

Our findings confirm these challenges, revealing limited preparedness among intermediaries and parents, as well as notable knowledge gaps among prevention workers themselves. Professionals also reported a disconnect between scientific research and practical application. Research is often seen as inaccessible, overly theoretical, and hard to implement—prompting intermediaries and policymakers to rely on intuition rather than evidence-based strategies. This aligns with findings by Oliver et al. [43], who suggest that limited access to research and insufficient training contribute to this divide.

To address these issues, participants emphasized the need for better access to scientific insights. A frequently proposed solution was the development of a centralized platform that translates research into clear, practical resources for youth, parents, and professionals.

### 4.3. Holistic Approach

Participants emphasized the need for a holistic approach to gaming prevention, one that integrates gaming behavior with other related digital risks. Research highlights the strong overlap between gaming disorder and other forms of problematic internet use [44], reinforcing the call for integrated strategies. Our findings build upon this perspective, revealing how the absence of such integration undermines the effectiveness of prevention efforts. This aligns with Denoo et al. [45], who advocate for a systemic analysis of the digital ecosystem to better understand links between gaming and gambling-like behaviors. Participants stressed that ecosystem-based strategies are not abstract concepts but crucial for practical implementation.

While calling for integration, participants also pointed to a key complication: the mismatch between gaming prevention and substance abuse frameworks. By linking gaming prevention to substance abuse networks, it often remains isolated from broader digital health initiatives, limiting opportunities for a more integrated approach. Although some voices cautiously point to differences in the prevention of gaming disorder and substance abuse [46], the observations in our study diverge from much of the existing literature, which often models gaming disorder prevention on substance abuse strategies [47,48], despite important differences in their underlying dynamics. Participants warned that such associations reinforce stigma and overlook the distinct nature of gaming behavior. As a result, gaming prevention is often disconnected from broader digital health initiatives, limiting opportunities for integration. This suggests the need for tailored strategies, supported by dedicated networks attuned to the digital context. In this regard, the Netherlands offers a promising example: the Digital Balance Model—repeatedly mentioned by our interviewees—explicitly links digital well-being to gaming and provides concrete implementation tools [49].

### 4.4. Support for Implementation Strategies

Our findings reveal a significant gap in the accessibility and practical use of prevention methods. Although parental concerns about excessive gaming are already widespread [14], prevention strategies remain widely underused. Two main factors contribute to this: structurally, there is a lack of centralized, high-quality resources—such as tools, guidelines, and referral systems—and many professionals lack the skills to promote and apply prevention effectively, particularly among vulnerable groups. Practically, stigma, financial constraints, and logistical barriers restrict access to existing services. Participants expressed a clear need for accessible, evidence-based tools tailored to gaming-related issues. A centralized platform could consolidate validated resources, map regional support services, and foster professional collaboration. In parallel, low-threshold programs should be culturally sensitive, affordable, and accessible through local outreach. However, professionals often feel unsure about how to implement such strategies, indicating a need for structural support and training.

This “know-do gap” [50] illustrates a broader challenge in translating knowledge into practice. Although prevention professionals are highly skilled, they frequently lack the tools to support implementation. Implementation science offers a promising framework by adapting evidence-based interventions to real-world settings [51]. Surprisingly, its potential remains underexplored in the field of gaming prevention.

To bridge this gap, we propose establishing a government-supported network that unites policymakers, practitioners, researchers, and implementation experts. This network could guide intervention development, deliver systematic training, and support context-sensitive strategies to reach underserved populations. Future research should assess how implementation science can enhance engagement and effectiveness in gaming disorder prevention.

### 4.5. Increasing Parental Involvement

Participants emphasized that parental involvement is crucial for preventing and addressing gaming disorder, supporting findings by Coşa et al. [52], who identify it as a key protective factor. To strengthen their impact, parents must first become aware of their influential role. Many underestimate their ability to shape gaming behavior, assuming their influence declines during adolescence. Participants stressed the need to guide parents in adopting effective communication and mediation strategies. Bradt et al. [53] show that autonomy-supportive approaches—combining clear limits with open dialogue—are more effective than controlling methods. Prevention programs should help parents establish boundaries, maintain communication about online activities, and offer consistent guidance. Finally, participants underlined the importance of family cohesion. Strengthening familial bonds was seen as a powerful buffer against gaming-related problems [54].

### 4.6. A Socio-Ecological Framework for Gaming Disorder Prevention

Applying the SEM to our analysis reveals key barriers and opportunities within gaming disorder prevention. Prevention professionals frequently encounter challenges that extend beyond program content, including institutional constraints, public policy gaps, and community resistance. The SEM provides a structured approach to mapping these dynamics, enabling a more strategic alignment of prevention initiatives with their broader ecosystem. The perspectives and proposed solutions of prevention professionals in our study consistently align with the layers of the SEM, illustrating its potential to structure discussions on intervention strategies.

Our findings affirm the applicability of the SEM in structuring the prevention landscape, resonating with the work of Rubio-Valera et al. [55]. Their study showed that the substantial barriers faced by prevention workers can generally be categorized within the levels of the SEM, underscoring the necessity of addressing these levels comprehensively in practice.

Rather than viewing the SEM as a rigid framework, it should be understood as a versatile analytical tool that enables structured problem analysis. Instead of replacing existing theories or models, the SEM provides an integrative structure within which they can be synthesized.

### 4.7. Limitations

This study has several limitations. First, voluntary participation may have introduced selection bias: individuals with stronger interest or involvement in (problematic) gaming could have been more likely to participate, potentially narrowing variation in literacy and engagement. We partly mitigated this by recruiting across prevention roles with different degrees of day-to-day contact with gaming prevention, yet a broader professional cross-section would further strengthen diversity. Second, this study explored both problematic gaming prevention and gaming-gambling convergence, with the interview guide (see Supplementary Materials) covering both topics. Because participants were aware of this dual focus, gambling-related themes may be over-represented. Third, one researcher’s involvement in an early-intervention network may have shaped data generation or interpretation. Fourth, because we did not conduct a cross-country mapping of prevention infrastructures or policy responses, transferability of the present Flemish findings should be interpreted cautiously. Even so, several barriers reported here—such as fragmented responsibilities, discontinuous resourcing, and gaps between a growing evidence base and the availability of well-evaluated, low-threshold tools—mirror concerns in international reviews and policy work on public-health responses to gaming-related harms [18,54,55,56]. Finally, within Belgium, we did not systematically assess differences between Dutch- and French-speaking contexts, although some interviewees held roles spanning both.

Future work should purposively sample across degrees of involvement in gaming prevention and explore the applicability of the proposed framework in other socio-cultural and institutional contexts.

## 5. Conclusions

This study demonstrates that effective prevention of problematic gaming requires a shift from individually focused interventions toward a systemic, multi-layered approach grounded in the SEM. By incorporating the voices of Flemish prevention professionals, actors who operate across interpersonal, institutional, and public policy levels, this research sheds light on the structural, relational, and contextual barriers that hinder current prevention efforts.

The findings underscore that the current policy landscape remains fragmented and reactive, lacking both the coordination and resources needed to meet the rising demand for support. Additionally, the disconnection between academic research and practical tools limits the capacity of professionals and parents to act preventively. Crucially, participants advocated for an integrated digital well-being approach, rather than isolated gaming prevention, to address the realities of young people’s interconnected online behaviors.

This study is the first to apply the SEM framework to gaming disorder prevention in practice, mapping out challenges and proposed solutions across all ecological layers. It empirically identifies the implementation gap between research, policy, and practice; furthermore, it emphasizes the central role of prevention professionals as system-bridging agents; and lastly, it proposes a roadmap for more coordinated and context-sensitive intervention strategies.

In doing so, this work offers concrete implications for policy and practice. Policymakers are encouraged to invest in long-term, multi-level strategies that align public health, education, and digital policy. Practitioners would benefit from accessible, evidence-informed resources and training grounded in the lived realities of youth and families. Future research should build on these findings by exploring how implementation science can help bridge the gap between knowledge and practice, and by testing the scalability of ecosystem-based prevention models in diverse contexts.

Ultimately, reframing gaming disorder prevention through a socio-ecological lens shifts the focus from individual responsibility to collective action—laying the groundwork for more sustainable and coordinated interventions.

## Data Availability

The data presented in this study are available on reasonable request from the corresponding author. The data are not publicly available due to the sensitive and potentially identifiable nature of the information.

## Supplementary Materials

The following supporting information can be downloaded at: https://www.mdpi.com/article/10.3390/ijerph23010117/s1, Supplementary Material S1: Semi-structured interview guide; Supplementary Material S2: Participant overview.
