# Supplementary material for: Rethinking Gaming Disorder Prevention: A Socio-Ecological Model Based on Practitioner Insights
Source: Int J Environ Res Public Health. 2026 Jan 17;23(1):117. doi: 10.3390/ijerph23010117 (PMC12841167; doi:10.3390/ijerph23010117)
Supplement: Supplementary file 1 [file ijerph-23-00117-s001.zip › ijerph_supplementary_S1_interview_guide.pdf]

<u>**Supplementary Material S1. Semi-structured interview guide**</u>

**Questions for Stakeholders on the Prevention of Problematic Gaming and Gambling-Game Convergence**

**Defining the Issue**

1. How do you define problematic gaming?
2. To what extent do you encounter this issue in your professional practice?
3. What do you understand by gambling-game convergence?
4. How frequently do you come across this phenomenon in your work?

**Current Prevention Efforts**

1. How is the prevention of risky gaming behavior currently addressed within your organization, and who is responsible for it?
2. What materials are currently used by different prevention workers? (Allow open responses first, then specify)
    - Informational brochures
    - Webinars
    - Training sessions for parents
    - Training sessions for gamers
    - Training sessions for frontline professionals
    - Early intervention initiatives
    - Other activities
3. Has your organization developed its own prevention materials?
4. Would you be willing to share these resources with us?
5. Does the topic of gambling-game convergence already feature in your materials? Why or why not?

**Target Groups and Needs**

*Youth*

1. Which groups of young people should be prioritized in prevention efforts of gaming and gaming/gambling convergence?

2. What specific needs do young people have in terms of prevention?
3. What strategies and environments should be used to engage young people effectively?
4. What should you be mindful of when designing prevention strategies for young people?
5. If you could hire someone for two years to work on preventing problematic gaming among young people, what would their role entail?
6. Should a prevention tool on gambling-game convergence be narrowly focused, or should it address a wider range of related issues?

*Parents*

1. Which groups of parents should be prioritized in prevention efforts?
2. What specific needs do parents have in terms of prevention?
3. What strategies and environments should be used to engage parents effectively?
4. What should you be mindful of when designing prevention strategies for parents?
5. If you could hire someone for two years to work on preventing problematic gaming among parents, what would their role entail?
6. Should a prevention tool on gambling-game convergence be narrowly focused, or should it address a wider range of related issues?

*Other Stakeholders*

1. Which stakeholders should be involved in prevention efforts?
2. Who do you consider to be the most important stakeholders in this field?
3. What specific needs do prevention workers have when engaging with stakeholders?
4. What strategies should be employed to engage stakeholders effectively?
5. What should we be mindful of when developing prevention strategies for stakeholders?
6. If you could hire someone for two years to work on preventing problematic gaming among parents, what would their role entail?
7. Should a prevention tool on gambling-game convergence be narrowly focused, or should it address a wider range of related issues?
