# Supplementary material for: Rethinking Gaming Disorder Prevention: A Socio-Ecological Model Based on Practitioner Insights
Source: Int J Environ Res Public Health. 2026 Jan 17;23(1):117. doi: 10.3390/ijerph23010117 (PMC12841167; doi:10.3390/ijerph23010117)
Supplement: Supplementary file 1 [file ijerph-23-00117-s001.zip › ijerph_supplementary_S2_participant_overview.pdf]

Supplementary Material S2. Participant overview

Participant ID	Role/function	Work Setting	Years of experience
R01	Prevention, Early Intervention Worker & Content creator	Mental Health Care Center	17
R02	Prevention Worker	Mental Health Care Center	18
R03	Prevention & Early Intervention Worker	Mental Health Care Center	7
R04	Prevention & Early Intervention Worker	Mental Health Care Center	5
R05	Prevention & Early Intervention Worker	Mental Health Care Center & Municipal Organization	14
R06	Prevention worker & Expertise promotor	Mental Health Care Center	15
R07	Prevention & Early Intervention Worker	Municipal Organization	4
R08	Prevention & Early Intervention Worker	Mental Health Care Center	6
R09	Media Literacy Coordinator	NGO	8
R10	Prevention & Early Intervention Worker	Mental Health Care Center	8

R11	Early Intervention Worker	Mental Health Care Center	5
R12	Prevention & Early Intervention Worker	Mental Health Care Center	22
R13	Prevention & Early Intervention Worker	Mental Health Care Center	4
R14	Prevention Worker, Early Intervention Worker & Content creator	Mental Health Care Center & Municipal Organisation	21
R15	Prevention & Early Intervention Worker	Mental Health Care Center	7
R16	Prevention Specialist & Tool Developer	NGO	8
R17	Prevention Specialist & Tool Developer	NGO	13
R18	Policy-level Prevention Professional	Municipal Organization	3
